# Development and standardization of the “Let's Shop” questionnaire: an assessment of shopping habits and executive functions in people with obesity

**DOI:** 10.1002/fsn3.412

**Published:** 2016-08-05

**Authors:** Sarit Akerman Frid, Naomi Josman, Ronit Endevelt

**Affiliations:** ^1^ School of Public HealthUniversity of HaifaHaifaIsrael; ^2^ Department of Occupational TherapyFaculty of Social Welfare & Health SciencesUniversity of HaifaHaifaIsrael; ^3^ Director of Health Promotion and Preventive Medicine Maccabi Health Medical Services and Head of Health Promotion track in the School of Public HealthUniversity of HaifaHaifaIsrael

**Keywords:** Executive functions, impulsivity, obesity, planning, shopping

## Abstract

Overweight and obesity are epidemic in Western countries and the literature suggests a relationship between overweight and executive functions (EF). Shopping is a regular, everyday activity that is strongly related to executive functioning. To date, no assessment tool has been developed to evaluate EF in adults with overweight and obesity, with a focus on the activity of shopping. To determine the factorial validity of the newly developed “Let's Shop” questionnaire. A convenience sample which included three groups of 93 men and women aged 35–60 were included in the study. Thirty three overweight participants and 30 obese participants who sought a clinical dietitian constituted the two research groups, and 30 normal weight participants recruited from a physician's waiting room constituted the control group and were matched to the two research groups by age, gender, education, and socioeconomic status. The “Let's Shop” questionnaire was administered to all participants. An exploratory principal factor extraction with oblimin rotation was conducted for the “Let's Shop” questionnaire. Twenty‐one items were entered into the equation; the analysis revealed four distinct factors comprised of 17 items. The cumulative percentage of variance accounted for by the four factors was 44.74%. The four factors were as follows: “planning in action” α = 0.63; *“*planning ahead” α = 0.71; *“*impulsivity” α = 0.67; and “habits” α = 0.63. The “Let's Shop” questionnaire was reduced to 17 items. This brief questionnaire will enable rapid administration by researchers and practitioners and determine a potential association between EF in the supermarket arena and weight status.

## 1 Introduction

In the last 30 years, overweight and obesity have increased to epidemic proportions in Western countries and have become one of the most common problems encountered in primary healthcare. Epidemiological research suggests that obesity and overweight are the main risk factors for diabetes, heart and blood diseases, cancer, and early death (Kelly, Yang, Chen, Reynolds, & He, 2008). The mechanism behind overweight is complicated and includes factors such as overeating, inadequate physical activity, genetics, endocrinology, behavioral, and cultural factors, as well as societal norms, which promote these phenomena (Hill, Wyatt, Reed, & Peters, 2003). In recent years, the literature has begun to suggest a relationship between overweight and executive functions (EF) (Boeka & Lokken, 2008; Lokken, Boeka, Austin, Gunstad, & Harmon, 2009; Maayan, Hoogendoorn, Sweat, & Convit, 2011; Willeumier, Taylor, & Amen, 2011). EFs are defined as higher‐order functions needed for performing complex or nonroutine tasks (Godefroy, 2003). They include (1) forming, (2) maintaining, and (3) shifting mental sets, corresponding to the ability to (1) reason and generate goals and plans, (2) maintain focus and motivation to follow through with those goals and plans, and (3) flexibility to alter those goals and plans in response to changing contingencies (Suchy, 2009). Dysexecutive syndrome refers to a collection of deficits in attention, planning, problem‐solving, multitasking, monitoring, and behavioral control (Burgess, Veitch, de Lacy Costello, & Shallice, 2000). People who suffer from impairments in EF typically have difficulty initiating or suspending activities and show impaired mental flexibility, and increased distractibility (Anderson & Tranel, 2002).

Although the recent literature shows a relationship between obesity and EF difficulties, the assessment tools that have been used for measuring EF were computerized with low ecological validity. EF are usually measured using neuropsychological tests; although these tests provide important information about impairments, they have low ecological validity. Hence, their ability to predict everyday functioning is limited (Chaytor, Schmitter‐Edgecombe, & Burr, 2006).

Shopping is considered to be one of the major everyday activities that most people perform. While shopping in the supermarket, many EF components control performance including planning, problem‐solving, categorizing, and inhibition. Since difficulties in EF can influence the ability to carry out and the efficiency of shopping, this activity was chosen as the focus of this study. No questionnaire or assessment tool for shopping habits was identified in the literature therefore, the “Let's Shop” tool was developed for use in this study. The purpose of this study was to describe the development of the “Let's Shop” questionnaire as an assessment tool for shopping habits and EF among people with a range of BMI (normal weight, overweight, and obese) and to report on the respective reliability and factor analysis validity. The purpose of the questionnaire is to evaluate relevant shopping habits and behaviors of people with a range of BMIs. The objectives of the current study were as follows:

1. To examine the questionnaire by expert validation
2. To develop construct validity of the questionnaire by factor analysis, and assess the questionnaire's internal consistency.

## 2 Methods

### 2.1 Subjects and design

**Figure 1:**
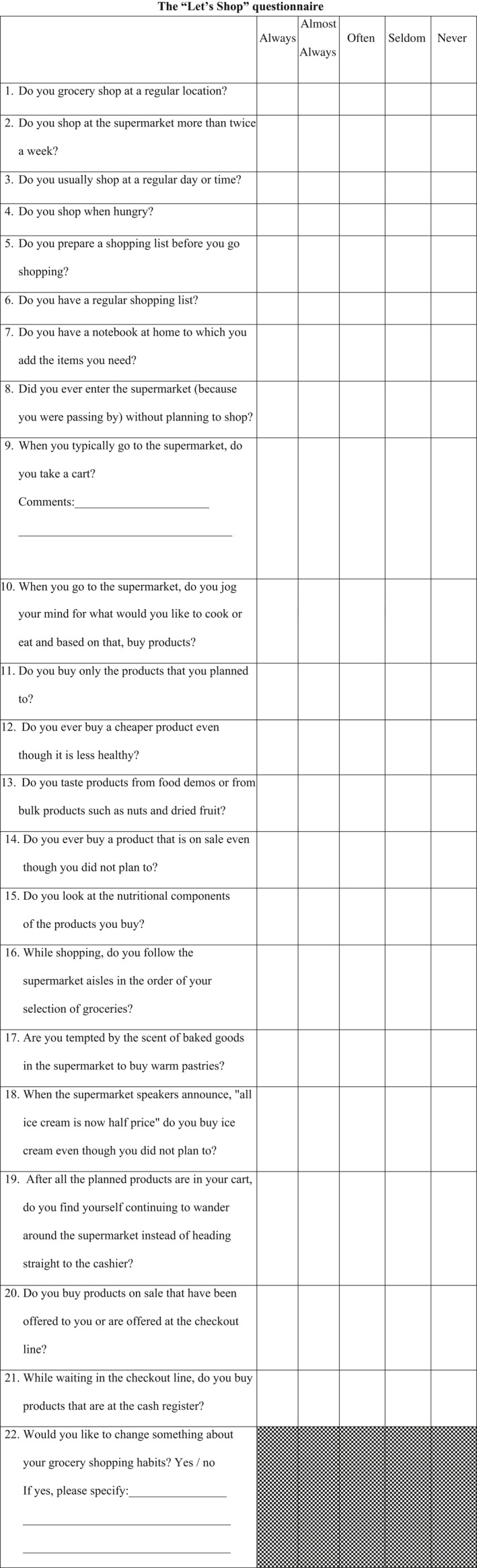
The “Let's‐Shop” questionnaire to assess shopping habits and executive function

Ninety‐three men and women aged 35–60 completed the “Let's Shop” questionnaire (Fig. 1) as part of a larger study that examined the relationships between EFs and different categories of BMI. The study included 33 overweight participants and 30 obese participants who visited a clinical dietitian at Maccabi Health Services. In addition, a control group of 30 participants with normal weight recruited from a physician's waiting room in the same clinic was included and matched for age, gender, education, and socio‐economic status. The study was approved by Maccabi Health Services Helsinki committee (approval number 04\2011), and all participants provided written informed consent. Demographics on gender, age, race/ethnicity, education, and income were also collected via questionnaire, and all participants completed the “Let's Shop” questionnaire. The following results relate to the development of the questionnaire and its reliability and validity**.** No significant differences between the groups were identified with respect to age (44.9 ± 7.9 years for the control group, 45.2 ± 7.5 years for the overweight group and 46.7 ± 8 for the obese group). In order to get information about level of income, the following question was developed: “The average household income in Israel is around 10,000 ₪. Is your income: (1) Much below average (2) Slightly below average (3) Around average (4) Slightly above average (5) Much above average. Additional participant socio‐demographic characteristics are presented in Table 1.

**Table 1:** Sociodemographic characteristics of participants by group

	*Groups*						χ^2^	*p*
	Obese *n*=30		Overweight *n*=33		Normal weight *n*=30			
	*n*	%	*n*	%	*n*	%		
Gender								
Male	12	42.9	11	39.3	5	17.9	4.13	.13
Female	18	27.7	22	33.8	25	38.5		
Education Level								
Elementary school	1	50	1	50	0	0	3.45	.75
High school	4	25	8	50	4	25		
Vocational/Technical school	8	30.8	10	38.5	8	30.8		
College	16	33.3	14	29.2	18	37.5		
Income								
Much below average	2	33.3	2	33.3	2	33.3	4.66	.79
Slightly below average	1	8.3	6	50	5	41.7		
Around average	7	28	9	36	9	36		
Slightly above average	12	41.4	9	31	8	27.6		
Much above average	7	35	7	35	6	30		
Occupation								
Working	27	31.8	31	36.5	27	31.8	2.04	.73
Unemployed	1	33.3	1	33.3	1	33.3		
Other	0	0	1	33.3	0	66.7		

### 2.2 The “Let's Shop” questionnaire

#### 2.2.1 The questionnaire development process

An occupational therapist and two dietitians performed a task analysis of shopping in the supermarket, which resulted in 26 questions. The questions were assessed by academic judges, nutrition and occupational therapy experts. The academic judges were requested to check whether each question was related to the topic of supermarket shopping habits, to which category it belonged (habits/planning/impulsiveness) and if there were other questions they felt should be added. Based upon the comments of the academic judges, any disagreed upon questions were removed, such as: “do you shop while speaking on your cell phone?” In addition, some questions shifted categories, for example, question 15, “do you typically look at the nutritional profile of products you purchase?” was moved from the planning category to the habits category, based upon the recommendation of the majority of the judges. Likewise, questions were added based upon their recommendation, such as question 2: “do you typically conduct your supermarket shopping more than once a week? After all the changes were implemented, the final version of the questionnaire contained 22 questions.

#### 2.2.2 Description of the questionnaire

The 22‐item questionnaire was administered to all participants. Participants were required to select a response for each item based upon on a 5‐point Likert scale (e.g. never, seldom, often, almost always and always). This scale relates to all questions, except the last, in which the scale is a “yes” or “no.” Therefore, only 21 items were included in the factor analysis (see Fig. 1).

### 2.3 Data analysis

Statistical analyses were performed using statistical analysis software (SPSS version 19.0 for Windows, 2010, SPSS Inc). Descriptive statistics were performed, nominal socio‐demographic findings were described according to their frequency in the sample, and the relationship between these variables and the research groups was examined using the chi‐square correlation coefficient. For continuous socio‐demographic variables, means, standard deviations, and *F* values were calculated. Afterwards, a factor analysis was performed with the “Let's Shop” questionnaire to determine its categories. In addition, a reliability internal consistency test was performed using the Cronbach Alpha test for all items in the “Let's Shop” questionnaire. Pearson's correlation was performed in order to determine the relationships among the factors obtained. In order to examine the existence of differences between four categories of the questionnaire among the participants, a MANOVA test was performed.

## 3 Results

### 3.1 Phase 1: Construction of the questionnaire and expert validation

The initial questionnaire, constructed by the researchers, was examined by academic experts, including occupational therapists (OTs) and clinical dietitians, who were asked to examine the relevance of each item to supermarket shopping habits for the evaluation of the questionnaire. If an item was deemed irrelevant, they were asked to explain why, to which category (habits, planning or impulsivity) the item was thought to be related and if there were other items they recommended adding. The questionnaire initially included 26 items, and was reviewed by three expert OTs. For 15 of the questions (75%–100%), the inter‐rater agreement percent among judges was very high, and there was 0% agreement regarding three items in the questionnaire. Therefore, in response to the judges' feedback, changes were made accordingly, new questions were added and controversial ones were removed. The questionnaire, which previously included 22 items, was then reexamined by other judges, including three nutrition and OT experts. The inter‐rater agreement percentage was 78%–100% regarding 11 items in the questionnaire, and 0%–67% for the other items. Hence, no additional changes were made to the questionnaire. Question number 22 (“would you like to change something about your shopping habits?”) was not included in the overall analysis of the questionnaire, since it did not belong to any of the questionnaire's categories, but rather is a qualitative question that examines the subject's awareness regarding his shopping habits, and requires a “yes” or “no” answer.

### 3.2 Phase 2: Examination of the questionnaire's validity and reliability

#### 3.2.1 Construct validity

An exploratory principal factor extraction with oblimin rotation was conducted for the “Let's Shop” questionnaire, to determine its factors. A factor loading above 0.35 was considered acceptable. Twenty‐one items were entered into the equation**;** the analysis revealed four distinct factors with Eigenvalues >1, comprised of 17 items (see Table 2). The cumulative percentage of variance accounted for by the four factors was 44.74%. The four factors were as follows:

**Table 2:** Exploratory factor analysis: 17 items from the “Let's Shop” questionnaire

Item	Item^a^	1 Planning in action	2 Planning ahead	3 Impulsivity	4 Habits
1	Do you grocery shop at a regular location?				0.612
3	Do you usually shop at a regular day or time?				0.616
4	Do you shop when hungry?			0.635	
5	Do you prepare a shopping list before you go shopping?		−0.768		
6	Do you have a regular shopping list?		−0.355		
7	Do you have a notebook at home to which you add the items you need?		−0.720		
8	Did you ever enter the supermarket (because you were passing by) without planning to shop?			0.410	
11	Do you buy only the products that you planned to?	0.381			
12	Do you ever buy a cheaper product even though it is less healthy?			0.447	
13	Do you taste products from food demos or from bulk products such as nuts and dried fruit?			0.441	
14	Do you ever buy a product that is on sale even though you did not plan to?	0.464			
15	Do you look at the nutritional components of the products you buy?	0.468			
16	While shopping, do you follow the supermarket aisles in the order of your selection of groceries?				0.497
17	Are you tempted by the scent of baked goods in the supermarket to buy warm pastries?	0.407			
18	When the supermarket speakers announce, “all ice cream is now half price” do you buy ice cream even though you did not plan to?	0.758			
19	After all the planned products are in your cart, do you find yourself continuing to wander around the supermarket instead of heading straight to the cashier?			0.437	
20	Do you buy products on sale that have been offered to you or are offered at the checkout line?			0.624	
	Eigenvalue	18.66	10.85	8.57	6.65
	% of variance	3.92	2.28	1.8	1.39
	Internal consistency (α)	0.63	0.71	0.67	0.63

a Items with factor loading<0.35 were omitted.

1. The first factor, “planning in action,” included five items and accounted for 18.66% of the variance with α = 0.63.
2. The second factor, “planning ahead,” included three items and accounted for 10.85% of the variance with α = 0.71.
3. The third factor, “impulsivity,” included six items and accounted for 8.57% of the variance with α = 0.67.
4. The fourth factor, “habits,” included three items and accounted for 6.65% of the variance with α = 0.63.

#### 3.2.2 Internal consistency

Cronbach's alpha coefficient was calculated for all 17 items; an alpha coefficient of 0.76 was found to indicate a satisfactory internal consistency. The Cronbach's alpha reliability values of each of the “Let's Shop” questionnaire factors are presented in Table 2.

#### 3.2.3 Correlations among factors

Pearson correlation was computed to evaluate the relationship between the factors. The results are presented in Table 3. As seen in Table 3, there were significant correlations between all the factors of the questionnaire and the correlation between “planning ahead” and “impulsivity” (r = .20) was found to be significant (*p* = .06).

**Table 3:** Correlations between the four factors of the questionnaire

	Planning ahead	Impulsivity	Habits
Planning in action	0.24^a^	0.28^b^	0.23^a^
Planning ahead		0.20^c^	0.43^b^
Impulsivity			0.21^a^

a *p *<* *.05.

b *p *<* *.01.

c *p *=* *.06.

An investigation of the differences among the three different BMI groups via MANOVA did not reveal significant differences. In addition, the answer “yes” to the qualitative question number 22 was not significantly different between the groups (*p *=* *.29). However, an interesting and significant result was found when examining the difference between males and females in the “planning ahead” category, in which the female group received a higher score (9.85) than the male group (7.92) (*F* (4,88 = 0.90 *p *<* *.05).

## 4 Discussion

Obesity is often explained as the result of a lack of control over nutrition and food‐related behavior. For many years, developing tailored treatment tools for best interventions proved to be a challenge. As obesity has now become an epidemic, there is a clear need for an easy‐to‐use tool for exploring the differences between people who suffer from the same problem and shopping in the supermarket is a good scenario in which to examine the issue. Due to the complexities of undertaking research in a real supermarket, there are studies that used a virtual supermarket environment (Waterlander, Jiang, steenhuis, & Ni Mhurchu, 2015) to investigate the shopping behavior of obese individuals. In this study, a questionnaire was developed which could be used to learn more about the participant's supermarket shopping experience and to investigate both the individual's shopping habits and EF while shopping. Furthermore, there is great potential for to expand knowledge about this daily habit by using such a self‐report questionnaire as it sheds light on the individual's perspective on his role and contribution to his or her condition (Rosenblum, Josman, & Togila, unpublished data).

Since the supermarket arena contains available food with a tempting appearance, planning, as well as other EF components are needed in order to ensure the purchase of healthy foods and to maintain appropriate nutrition and weight (Vinkers, Adriaanse, Kroese, & de Ridder, 2014). The aims of this study were to develop a questionnaire to investigate the EFs of three BMI category groups in the supermarket arena, to develop construct validity of the questionnaire by factor analysis, and to report on the questionnaire's internal consistency. In the first phase of the study, the questionnaire was tested for expert validation by specialists from two different professions: occupation therapists who are experts in executive function, and dietitians, to better understand supermarket behavior. On the basis of the two professions' knowledge, the items included in the questionnaire were conceptually grouped into three categories: planning, impulsivity and habits. However, following the factor analysis, the planning category was divided into two distinct categories: “planning in action” and “planning ahead.” The internal consistency of each category was found to be satisfactory despite the relatively small number of participants in the study.

The four categories consist of the following: (1) planning in action within the supermarket, where plenty of foods are available, (2) planning ahead skills, such as writing a list for shopping, bringing it to the supermarket and purchasing only what was planned (crucial for the success of any nutritional behavior to for weight maintenance) (3) impulsivity**,** considered to be typical for obese individuals facing the temptation of food and (4) habits**,** activities performed on a daily basis without planning and thinking ahead. The questionnaire possessed satisfactory internal consistency and close to a significant positive correlation between “planning ahead” and “impulsivity,” therefore, it might be possible, that the correlation would be significant in a larger sample. As shown in other studies, self‐regulation and planning ahead are the opposite trait of impulsivity (Miller et al., 2012).

A recent study found that higher BMI is associated with decreased inhibitory control over food‐related responses, and not characterized by a general tendency to react impulsively but rather by an impulsive response toward palatable food (Houben, Nederkoorn, & Jansen, 2014). Since impulsive buying is the opposite of planning ahead, the correlation found in our study was positive because the “impulsivity” category included negative questions that were inverted in same direction of all questions in the questionnaire.

Furthermore, in this study, we expected to find differences between the BMI categories for normal weight versus overweight and obesity in the four categories of the questionnaire. However, as opposed to the literature, no significant differences were found. This result may be explained by the fact that the study was a convenience sample of people waiting to receive nutritional counseling, thus it is possible that this population had better executive function skills compared to the general overweight population. It may also be that since dietitians offer support for patients regarding temptation control and planning ahead in purchasing food, this skill may be better developed in these participants.

The questionnaire was tested later for variance in planning in action, planning ahead, impulsivity and habits, and a significant difference was found between men and women in the planning ahead category. It is important to note that this category included only the following three items: (1) do you prepare a shopping list before you go shopping? (2) do you have a regular shopping list? and (3) do you have a notebook at home to which you add the items you need? Interestingly, women had a greater tendency to plan ahead than men, but did not exhibit higher marks than men on planning within the supermarket, habits, or impulsivity. This finding is supported in the literature by studies which found that women tend to buy more healthy products with better awareness then men (Hardin‐Fanning & Gokun, 2014). In addition, neuroimaging studies identified gender differences in neural responses underlying planning. While women use more executive strategies for planning, men tend to rely more on spatial reasoning (Boghi et al., 2006). Developing a diagnostic tool for overweight and obesity treatment is important for planning the proper intervention in the tempting supermarket arena and other environment hazards.

### 4.1 Limitations

The study group in each BMI category was small, which may have influenced the results. Larger groups in each BMI category might have revealed significant differences in the scores that represent the behavior of the different BMI category consumers. The fact that the group, as mentioned above, was homogenous, from the same geographical area, and that obese and overweight participants received nutritional treatment may have influenced the responses of the participants from a conformity point of view. In addition, the group included participants whose income was above average (see Table 1). In this study, we focused on building the factor analysis validity of the questionnaire developed.

## 5 Conclusions

The brief, 17‐item “Let's Shop” questionnaire will enable researchers and practitioners the possibility of gaining knowledge regarding possible associations between EF and weight status, as well as the ability to better establish goals and objectives for intervention within this population. Further studies are required with larger, heterogeneous samples to investigate both the differences between the groups and to discover whether EF constitutes an underlying mechanism of shopping behaviors of people with overweight and obesity. In addition, following this study, which revealed the factorial validity of the “Lets‐shop” questionnaire, future research should focus on the development of construct validity by establishing its correlation with other known neuropsychological EF assessment tools.

## Funding Information

No funding source is involved in this report.

## Conflict of Interest

The authors state that there is no conflict of interest regarding this work.
